# Chromatin Separation Regulators Predict the Prognosis and Immune Microenvironment Estimation in Lung Adenocarcinoma

**DOI:** 10.3389/fgene.2022.917150

**Published:** 2022-07-08

**Authors:** Zhaoshui Li, Zaiqi Ma, Hong Xue, Ruxin Shen, Kun Qin, Yu Zhang, Xin Zheng, Guodong Zhang

**Affiliations:** ^1^ Qingdao Medical College, Qingdao University, Qingdao, China; ^2^ Cardiothoracic Surgery Department, Qingdao Hiser Hospital Affiliated to Qingdao University, Qingdao, China; ^3^ Heart Center Department, Qingdao Hiser Hospital Affiliated to Qingdao University, Qingdao, China; ^4^ Cancer Center Department, Qingdao Hiser Hospital Affiliated to Qingdao University, Qingdao, China; ^5^ Thoracic Surgery Department, Shandong Cancer Hospital Affiliated to Shandong First Medical University, Jinan, China

**Keywords:** lung adenocarcinoma, chromosome segregation regulators, prognostic signature, immune environment, bioinformatics

## Abstract

**Background:** Abnormal chromosome segregation is identified to be a common hallmark of cancer. However, the specific predictive value of it in lung adenocarcinoma (LUAD) is unclear.

**Method:** The RNA sequencing and the clinical data of LUAD were acquired from The Cancer Genome Atlas (TACG) database, and the prognosis-related genes were identified. The Kyoto Encyclopedia of Genes and Genomes (KEGG) and Gene Ontology (GO) were carried out for functional enrichment analysis of the prognosis genes. The independent prognosis signature was determined to construct the nomogram Cox model. Unsupervised clustering analysis was performed to identify the distinguishing clusters in LUAD-samples based on the expression of chromosome segregation regulators (CSRs). The differentially expressed genes (DEGs) and the enriched biological processes and pathways between different clusters were identified. The immune environment estimation, including immune cell infiltration, HLA family genes, immune checkpoint genes, and tumor immune dysfunction and exclusion (TIDE), was assessed between the clusters. The potential small-molecular chemotherapeutics for the individual treatments were predicted via the connectivity map (CMap) database.

**Results:** A total of 2,416 genes were determined as the prognosis-related genes in LUAD. Chromosome segregation is found to be the main bioprocess enriched by the prognostic genes. A total of 48 CSRs were found to be differentially expressed in LUAD samples and were correlated with the poor outcome in LUAD. Nine CSRs were identified as the independent prognostic signatures to construct the nomogram Cox model. The LUAD-samples were divided into two distinct clusters according to the expression of the 48 CSRs. Cell cycle and chromosome segregation regulated genes were enriched in cluster 1, while metabolism regulated genes were enriched in cluster 2. Patients in cluster 2 had a higher score of immune, stroma, and HLA family components, while those in cluster 1 had higher scores of TIDES and immune checkpoint genes. According to the hub genes highly expressed in cluster 1, 74 small-molecular chemotherapeutics were predicted to be effective for the patients at high risk.

**Conclusion:** Our results indicate that the CSRs were correlated with the poor prognosis and the possible immunotherapy resistance in LUAD.

## Introduction

Lung cancer is a malignant tumor with the highest mortality rate in the world (Nooreldeen and Bach, 2021). Lung adenocarcinoma (LUAD) is now the most common histological subtype of primary lung cancer (Travis et al., 2011; Torre et al., 2016; Hutchinson et al., 2019), accounting for more than 40% of cases (Hutchinson et al., 2019). Improvements in multimodal treatment strategies (e.g., targeted therapy, radiotherapy, and immunotherapy) have markedly increased the overall survival (OS) of LUAD-patients in recent years (Moreira and Eng, 2014; Jin et al., 2020), quite a few patients eventually become resistant to these therapies, partly attributed to the malfunctioning of genes that regulate cardinal bioprocesses (Philpott et al., 2017). Thus, sufficient strategies are needed to predict prognosis and guide individual treatment in LUAD. The availability of public cohorts with RNA sequencing data and improved technology brought the opportunity to identify a more generalized prognostic signature for LUAD. For instance, an immune-related four-gene prognostic signature in LUAD was identified to regulate the innate immune response and to be a benefit for the prognosis prediction (Sun et al., 2020). Pyroptosis-related prognostic gene signature and metabolism-associated gene signature were also identified as a predictor of the prognosis in LUAD-patients (He et al., 2020; Lin et al., 2021). However, the novel biomarkers with guiding significance for therapy of LUAD still need to be explored.

Mitotic cell division is commonly thought to involve the equal distribution of duplicated genomes into the two daughter cells through appropriate chromosome segregation (Neumüller and Knoblich, 2009; Sarkar et al., 2021). Abnormal chromosome segregation at mitosis causes the aneuploidy of the daughter cells with an unequal distribution of chromosomes (Levine and Holland, 2018), this is one way by which neoplastic cells accumulate the many genetic abnormalities required for tumor development (Gisselsson, 2008; Naylor and van Deursen, 2016; Levine and Holland, 2018). Moreover, mitotic errors and aneuploidization are found during tumor evolution, and the extent of chromosomal aberrations is correlated with tumor grade and poor prognosis (Loeper et al., 2001; M'Kacher et al., 2010; Bakhoum et al., 2011; Levine and Holland, 2018; Ben-David and Amon, 2020). Cells have well-conserved mechanisms to ensure proper chromosome segregation (Tanaka and Hirota, 2009), whose dysregulation may be involved in tumorigenesis. Therefore, chromosome missegregation is becoming a critical hallmark of tumor biology.

In this study, we determined a chromosome segregation-related gene prognostic signature from The Cancer Genome Atlas (TACG) LUAD cohort. The distinct chromosome segregation regulators (CSRs)-related clusters of LUAD-samples were established, and the overall survival (OS) and the immune environment estimation were assessed in the distinct clusters. This study indicates that the CSRs were correlated with the poor prognosis and the possible immunotherapy resistance in LUAD, which might lay a theoretical foundation for the individualized treatment of LUAD-patients.

## Materials and Methods

### Data Acquisition

The gene expression matrix (HTSEQ-Counts, HTSEQ-FPKM) for LUAD was acquired from the Genomic Commons Data Portal GDC (https://portal.gdc.cancer.gov/) of The Cancer Genome Atlas (TCGA) database (Tomczak et al., 2015), which contains 510 samples of patients and 58 normal samples. Clinical survival data (*n* = 738) and phenotype data (*n* = 877) of TCGA-LUAD matched patients were acquired from TCGA database. Excluding patients without information of survival, a total of 497 patients were retained for the prognostic analysis. For the nomogram model construction, 115 patients without information of the clinical index were excluded, and a total of 382 patients were retained.

The external validation sets of the Cox model were constructed using GSE3141 (*n* = 111) (Bild et al., 2006), GSE13213 (*n* = 117) (Tomida et al., 2009), GSE31210 (*n* = 226) (Yamauchi et al., 2012), GSE30219 (*n* = 278) (Rousseaux et al., 2013), and GSE50081 (*n* = 181) (Der et al., 2014) datasets from the Gene Expression Omnibus (GEO) database (Barrett et al., 2013). The data was acquired by the GEOquery R package (Zhou J. et al., 2021).

### The Cox and Nomogram Model Construction

The gene expression matrix of the 48 CSRs in the 497 LUAD samples was used for the univariate cox regression, LASSO regression, and multivariate cox regression analyses. The Survival R package was used to calculate the correlation between the expression of each gene and overall survival (OS), and genes with a *p*-value < 0.05 were retained for the following LASSO regression analysis. Glmnet and the survival R package were used for the LASSO regression analysis to screen the significant variables in the univariate cox regression analysis. In order to obtain more accurate independent prognostic factors (prognostic characteristic genes), multivariate cox regression analysis was used for the final screening. The risk score was calculated as the follows: risk score = (exp-gene1*coef-gene1) + (exp-gene n*coef-gene n). Patients were divided into high- and low-risk groups based on the median of risk score.

Time-dependent receiver operating characteristic (ROC) curves were used to assess survival predictions, and the Time ROC R package was used to calculate the area under the ROC curve (AUC) value to measure prognosis and predict accuracy. Survcomp R package was used for the C-index analysis. For the nomogram analysis, the phenotype data (*n* = 382) was used and the clinical indexes, including age, gender, race, TNM staging, and stage, were brought into the nomogram analysis. The calibration and decision curve analysis (DCA) were performed to assess the predictive power of the nomogram model.

The correlation of the 48 CSRs with the risk score was determined by Spearman correlation analysis. The Wilcoxon rank-sum test was used for the significant statistics.

### Identification of CSRs Pattern in LUAD Patients

Unsupervised clustering analysis (Testa et al., 2021) was used to identify the distinct clusters of LUAD patients according to the expression of 48 CSRs. R “Consensus Cluster Plus” package (Wilkerson and Hayes, 2010) was used for the clustering analysis.

### DEGs Determination and the Functional Enrichment Analysis

The differentially expressed genes (DEGs) were calculated using HTSEQ-FPKM of TCGA-LUAD by the Deseq 2 R package (Love et al., 2014), and visualized by the Ggplot 2 R package. The threshold is folded change>2 and *P*_*. adjust*_ <0.05.

Gene Ontology (GO) (Ashburner et al., 2000) and pathway Kyoto Encyclopedia of Genes and Genomes (KEGG) (Ogata et al., 1999) enrichment were performed by the ClusterProfiler R package (Yu et al., 2012), and visualized by the Ggplot 2 R package.

### Identification of Hub Genes

The protein-protein interaction network was constructed by STRING (https://cn.string-db.org/) (Szklarczyk et al., 2019), and visualized by Cytoscape (v3.7.2) (Shannon et al., 2003). The rank of each gene in the network was calculated by CytoHubba (Chin et al., 2014). The Top 50 hub genes were chosen for the following analysis.

### TME Estimate Analysis

Stromal score, Immune score, ESTIMATE score, and Tumor purity score were calculated based on mRNA expression matrix (Count, *n* = 497) by an estimate R package (Yoshihara et al., 2013). Immunization checkpoint block (ICB) assessment was performed by calculating the tumor immune dysfunction and exclusion (TIDE) score. This is a kind of computing algorithm based on gene expression profiles (http://tide.dfci.harvard.edu) (Fu et al., 2020). The difference between HLA family and immune checkpoint genes between the two clusters was performed based on the TPM of these genes.

The immune cell infiltration was calculated respectively by CIBERSORT algorithm (Newman et al., 2015) and xCell algorithm (Aran et al., 2017). The gene expression matrix data (FPKM, *n* = 497) were uploaded to CIBERSORT, and the 22 types of immune cell infiltration matrix were obtained. The distribution of the immune cell infiltration in each sample was shown using Ggplot 2 R package. The 38 types of cells in xCell algorithm were obtained by immunedeconv R package (Sturm et al., 2020).

The correlation of the 48 CSRs with immunocyte fraction was determined by Spearman correlation analysis. The Wilcoxon rank-sum test was used for the significant statistics.

### Correlation Analysis Between CSRs and TMB

For each tumor sample, the total number of somatic mutations (except silent mutations) detected in the tumor is defined as the tumor mutation burden (TMB) (Merino et al., 2020), TMB score for each sample was calculated, and the difference between the two clusters was performed. The correlation of the 48 CSRs with the TMB score was determined by Spearman correlation analysis. The Wilcoxon rank-sum test was used for the significant statistics.

### The Chemotherapeutics Forecast

The 50 hub genes were used for the chemotherapeutics forecast, which was performed using the mode of action (moa) module of the connectivity map (CMap, https://clue.io/command) (Gao et al., 2019).

### Statistical Analysis

The statistical analysis was calculated via Wilcoxon rank-sum test and unpaired t-text. All statistical tests were bilateral. All statistical tests and visualization were performed in R software (version 4.0.2).

## Results

### Chromatin Separation Is the Main Bioprocess Enriched by the Prognostic Genes in LUAD

**FIGURE 1:**
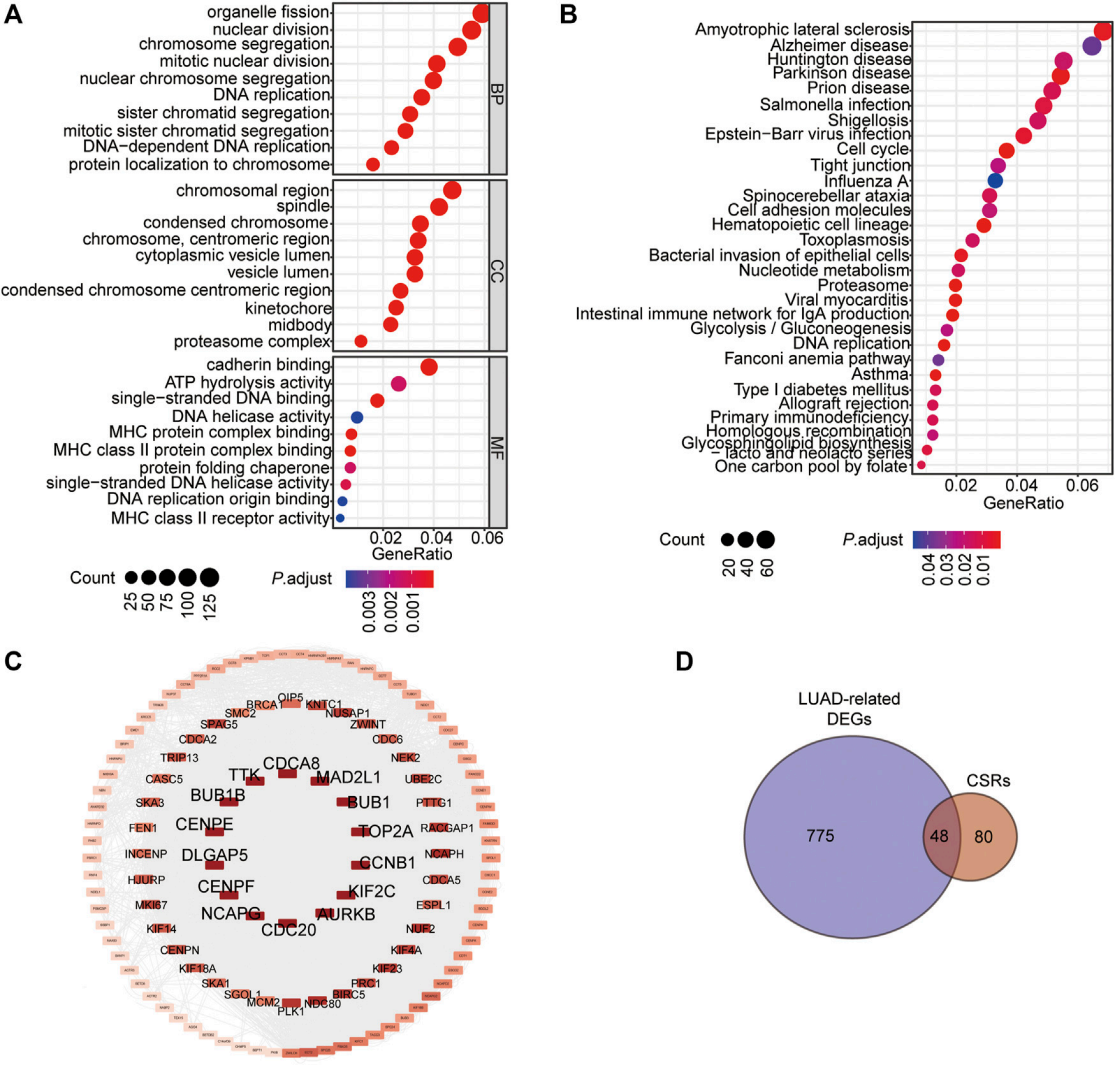
Chromatin Separation is the main bioprocess enriched by the prognostic genes in LUAD. **(A)** GO enrichment of the prognostic genes in LUAD. **(B)** KEGG enrichment of the prognostic genes in LUAD. **(C)** PPI network of the 128 CSRs. **(D)** Venn drawing showing the co genes between the 128 CSRs and LUAD-related DEGs. The LUAD-related DEGs were genes differentially expressed in LUAD-samples compared to the normal samples. Fold change>2, *P*. adjust<0.05.

To determine the significant genes involved in the prognosis of patients with LUAD, the batch prognostic analysis of whole genes in samples of TCGA-LUAD was performed. Among the 17,430 genes, a total of 2,416 genes significantly correlated with prognosis were obtained, with a threshold of *p*-value < 0.05 (Supplementary Table S1). GO and KEGG were performed to analyze the functional enrichment of these prognosis related genes, which showed that these genes were enriched in the biological processes of chromosome segregation, organelle fission, and nuclear division, the cellular components of chromosomal region, condensed chromosome, centromeric region, spindle, and condensed chromosome, and molecular functions of cadherin binding, single-stranded DNA binding, and ATP hydrolysis activity (Figure 1A, Supplementary Table S2). Additionally, the KEGG pathways these prognostic genes were enriched in Amyotrophic Lateral sclerosis, Parkinson's disease, Cell cycle, and DNA replication (Figure 1B, Supplementary Table S3). We noticed that chromosome segregation was the most significant bioprocess enriched by these genes (*P*.adjust = 5.5350E-19). We selected 128 genes enriched in the terms of chromosome segregation, nuclear chromosome segregation, and sister chromatid segregation for the subsequent analysis (Figure 1C, Supplementary Table S4), and we named them chromosome segregation regulators (CSRs). According to the calculated rank in the Protein-Protein interaction (PPI) network (Figure 1C), the top 14 genes were selected, including centromere protein E (*CENPE*), mitotic arrest deficient 2 like 1 (*MAD2L1*), BUB1 mitotic checkpoint serine/threonine kinase (*BUB1*), BUB1 mitotic checkpoint serine/threonine kinase B (*BUB1B*), TTK protein kinase (*TTK*), cell division cycle 20 (*CDC20*), aurora kinase B (*AURKB*), aurora kinase B (*KIF2C*), DNA topoisomerase II alpha (*TOP2A*), DLG associated protein 5 (*DLGAP5*), non-SMC condensing I complex subunit G (*NCAPG*), cyclin B1 (*CCNB1*), centromere protein F (*CENPF*), and cell division cycle associated 8 (*CDCA8*) (Figure 1C, Supplementary Table S5). Among these genes, a total of 48 CSRs were found to overlap with the 823 DEGs in TCGA-LUAD (Figure 1D, Supplementary Table S6, Supplementary Table S7).

The Kaplan-Meier analysis showed that the 48 CSRs were all correlated with the poor OS in LUAD (Supplementary Figure S1). In addition, the Spearman correlation analysis showed that these CSRs were positively associated with the high TMB in LUAD (Table 1). These indicated that the 48 CSRs may play important roles in LUAD progression.

**TABLE 1:** Correlation of CSRs with Risk_Score and TMB.

Gene Name	Risk_Score		TMB	
	Correlation (spearman)	*p*-Value	Correlation (spearman)	*p*-Value
AURKB	0.36	<2.2e-16	0.46	3.31e-27
BIRC5	0.41	<2.2e-16	0.43	2.04e-24
BUB1	0.45	<2.2e-16	0.45	5.99e-6
BUB1B	0.49	<2.2e-16	0.40	4.84e-21
CCNB1	0.48	<2.2e-16	0.38	2.92e-18
CCNE1	0.34	<4.4e-15	0.38	1.33e-18
CDC20	0.43	<2.2e-16	0.47	1.12e-28
CDC6	0.42	<2.2e-16	0.41	6.12e-22
CDCA2	0.46	<2.2e-16	0.38	8.74e-19
CDCA5	0.46	<2.2e-16	0.45	2.7e-26
CDCA8	0.41	<2.2e-16	0.45	8.53e-26
CDT1	0.38	<2.2e-16	0.37	6.67e-18
CENPE	0.45	<2.2e-16	0.39	7.37e-20
CENPF	0.43	<2.2e-16	0.40	6.8e-21
CENPK	0.36	<2.2e-16	0.30	1.53e-11
DLGAP5	0.5944	<2.2e-16	0.42	5.64e-23
EME1	0.26	2.8e-09	0.45	1.4e-26
ESPL1	0.3928	<2.2e-16	0.43	1.61e-23
FAM83D	0.43	<2.2e-16	0.36	2.19e-16
HJURP	0.5546	<2.2e-16	0.46	2.7e-28
KIF14	0.5192	<2.2e-16	0.43	8.08e-24
KIF18B	0.3933	<2.2e-16	0.43	2.17e-24
KIF23	0.5	<2.2e-16	0.44	5.55e-25
KIF2C	0.43	<2.2e-16	0.48	5.13e-30
KIF4A	0.45	<2.2e-16	0.40	4.16e-21
KIFC1	0.4	<2.2e-16	0.47	1.29e-28
KNTC1	0.21	3.2e-06	0.39	3.31e-19
MAD2L1	0.46	<2.2e-16	0.36	4.73e-17
MKI67	0.5	<2.2e-16	0.36	1.44e-16
NCAPG	0.46	<2.2e-16	0.43	1.49e-23
NCAPH	0.45	<2.2e-16	0.47	1.06e-28
NDC80	0.45	<2.2e-16	0.46	6.56e-26
NEK2	0.45	<2.2e-16	0.47	4.71e-29
NUF2	0.354	<2.2e-16	0.52	1.2e-34
NUSAP1	0.47	<2.2e-16	0.39	1.12e-19
OIP5	0.5075	<2.2e-16	0.40	5.67e-21
PLK1	0.6434	<2.2e-16	0.42	1.35e-22
PRC1	0.51	<2.2e-16	0.40	1.43e-20
SKA1	0.45	<2.2e-16	0.44	2.32e-25
SKA3	0.48	<2.2e-16	0.45	8.21e-27
SPAG5	0.38	<2.2e-16	0.46	9.98e-28
SPC24	0.35	2.3e-15	0.39	2.58e-19
SPC25	0.45	<2.2e-16	0.40	6.9e-21
TOP2A	0.38	<2.2e-16	0.45	6.78e-25
TRIP13	0.37	<2.2e-16	0.45	5.54e-27
TTK	0.41	<2.2e-16	0.47	1.48e-29
UBE2C	0.34	2.9e-15	0.47	1.04e-28
ZWINT	0.39	<2.2e-16	0.39	2.54–19

### The CSRs Are Involved in LUAD Process

**FIGURE 2:**
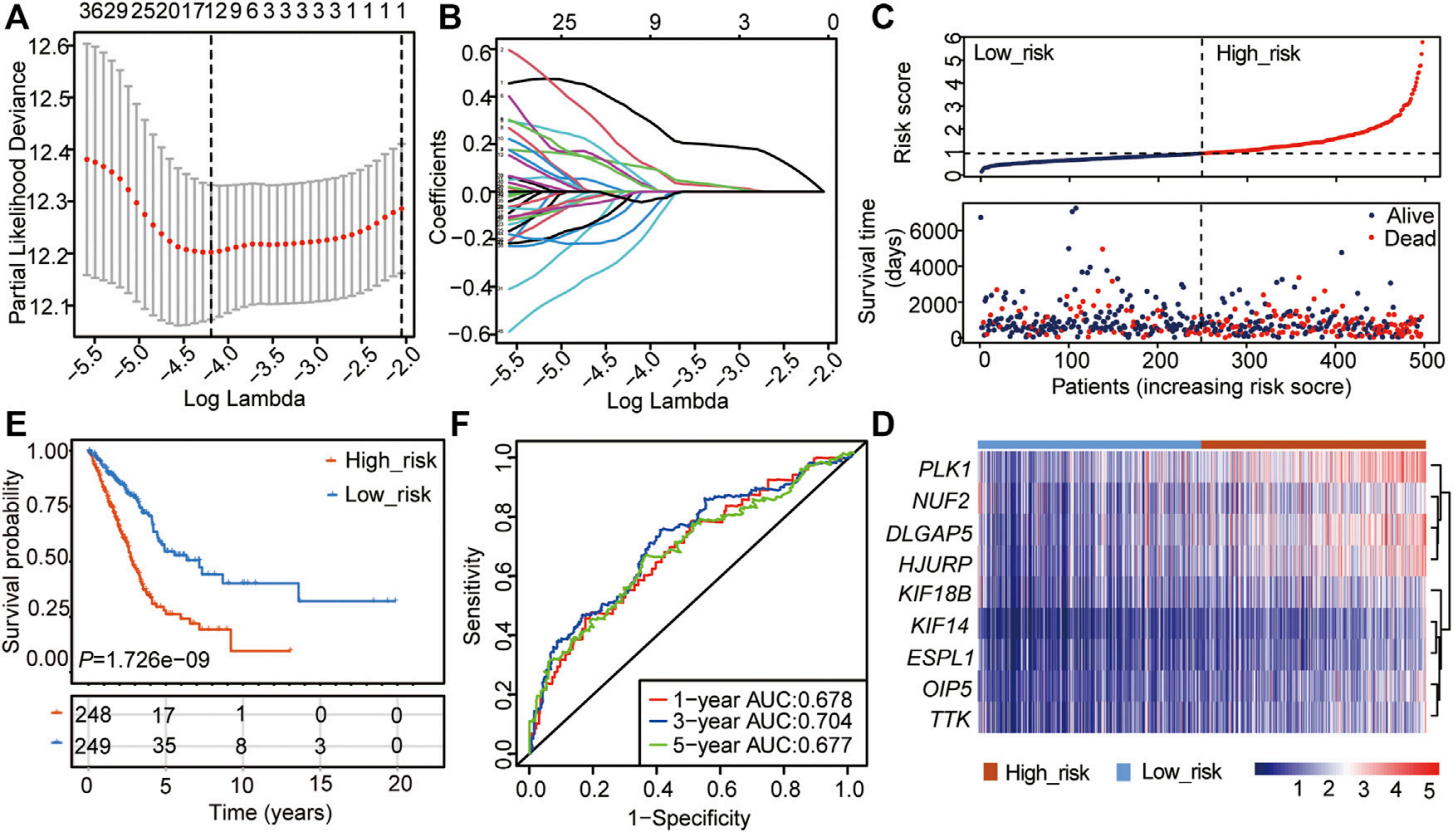
Construction of the cox regression model by the 48 CSRs. **(A)** LASSO coefficient spectrum of prognostic gene screening. **(B)** Stepwise Cox proportional risk regression model to screen the prognostic genes. **(C)** The risk score distribution and survival status of patients in the training cohort. **(D)** The heatmap of prognostic gene distribution in the training cohort. **(E)** The overall survival of high- and low-risk groups. **(F)** ROC analyses of the cox model for 1-, 3-, and 5-years.

The 48 CSRs we identified were used to perform the Univariate Cox regression analysis in TCGA-LUAD (*n* = 497), and 47 genes were eligible for screening (*p*-value < 0.05, Supplementary Table S8). The 47 genes were then chosen to perform the LASSO regression, and 12 genes were screened out to build a multivariate Cox regression analysis (Figures 2A,B, Supplementary Table S9). Finally, 9 genes, including *PLK1*, TTK, DLG associated protein 5 (*DLGAP5*), Holliday junction recognition protein (*HJURP*), kinesin family member 14 (*KIF14*), Opa interacting protein 5 (*OIP5*), extra spindle pole bodies like 1, separase (*ESPL1*), kinesin family member 18B (*KIF18B*), and NUF2 component of NDC80 kinetochore complex (*NUF2*), were identified to be independent prognostic signatures (Figures 2C,D, Supplementary Table S10, Supplementary Table S11). The 497 LUAD samples were divided into two subgroups with different risk scores: high- and low-risk subgroups, according to the median risk score based on the 9-gene independent prognostic signature (Figure 2C). The mortality of the high-risk group was higher than that of the low-risk group (Figure 2C), and the patients in the high group had a poor outcome compared with those in the low-risk group (*p* = 1.726e-09, Figure 2E). Meanwhile, the 48 CSRs were all positively associated with the risk score (Figure 2D; Table 1). The ROC curve was used to predict the prognosis for 1-year, 3-years, and 5-years, which showed that the AUC value ranged from 0.628 to 0.73 (Figure 2F). These indicated that the high expression of CSRs predicts the poor OS in LUAD.

### Construction of Nomogram Model by the Nine-Gene Prognostic Signature

**FIGURE 3:**
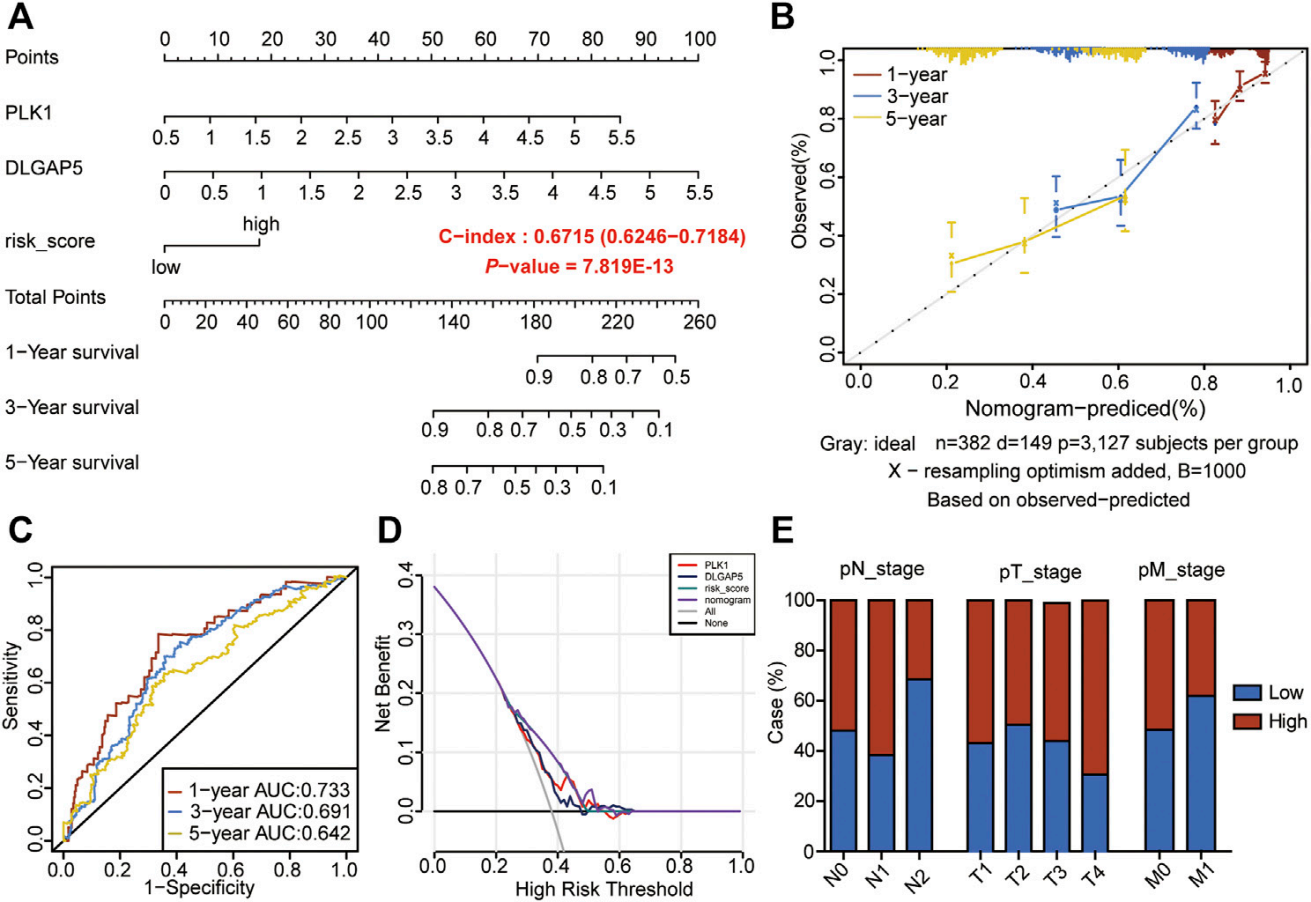
Construction of nomogram model by the 9-gene prognostic signature. **(A)** The nomogram to predict the prognosis of LUAD-patients. **(B)** The calibration analysis of the nomogram predicted a probability of 1-, 3-, and 5-years survival. **(C)** ROC analyses of the nomogram model for 1-, 3-, and 5-years. **(D)** DCA result of the prognostic model. **(E)** Risk groups in relation to a clinical index.

To further analyze the prognostic significance of the CSRs in LUAD patients, the 9-gene prognostic signature was used to perform the nomogram model with the combination of the clinical indexes, including pT_stage, pN_stage, pM_stage, and stage (Table 2). Two significant genes (*PLK1* and *DLGAP5*) were brought into the nomogram model, which showed that the expression values of the two genes and the risk score predicted the survival of LUAD patients (Figures 3A,B). The ROC curve showed that the nomogram Cox model could precisely predict the survival of patients, with the AUC ranging from 0.642 to 0.733 (Figure 3C). The decision curve analysis (DCA) and calibration analysis of nomogram predicted probability also suggested the accuracy of the Cox model (Figures 3B,D). However, there was no significant difference in the pTNM staging between the two risk groups (Figure 3E).

**TABLE 2:** The clinical index of LUAD patients used in the Cox model.

Characteristic	Levels	Overall	High-Risk	Low-Risk
n (Dead/Alive)		382 (149/233)	204 (103/101)	178 (46/132)
Age, n (%)	>=65	213 (55.76%)	114 (53.52%)	98 (46.48%)
	<65	169 (44.24%)	90 (44.12%)	90 (55.88%)
Gender, n (%)	Male	170 (44.50%)	78 (45.88%)	92 (54.12%)
	Female	212 (55.50%)	126 (59.43%)	86 (40.57%)
N stage, n (%)	N0	249 (65.18%)	129 (51.80%)	120 (48.20%)
	N1	65 (17.01%)	40 (61.53%)	25 (38.47%)
	N2	56 (14.65%)	27 (48.21%)	29 (51.79%)
	N3	1 (0.26%)	1 (100%)	0 (0)
	NX	11 (2.90%)	7 (63.63%)	4 (36.37%)
M stage, n (%)	M0	241 (63.08%)	124 (51.45%)	117 (48.55%)
	M1	21 (5.49%)	8 (38.09%)	13 (61.91%)
	MX	120 (31.43%)	72 (60.00%)	48 (40.00%)
T stage, n (%)	T1	132 (34.55%)	75 (56.82%)	57 (44.18%)
	T2	196 (51.30%)	97 (49.49%)	99 (50.51%)
	T3	40 (10.47%)	22 (55.00%)	18 (45.00%)
	T4	13 (3.40%)	9 (69.23%)	4 (30.77%)
	TX	1 (0.28%)	1 (100%)	0 (0%)
Pathologic stage, n (%)	Stage I	209 (54.71%)	107 (51.20%)	102 (48.80%)
	Stage II	87 (22.77%)	53 (60.92%)	34 (39.08%)
	Stage III	59 (15.44%)	33 (55.93%)	26 (44.07%)
	Stage IV	21 (5.49%)	8 (38.10%)	13 (61.90%)
	N/A	6 (1.59%)	3 (50.00%)	3 (50.00%)

### Validation of the CSRs-Related Prognostic Signature With GEO Datasets

**FIGURE 4:**
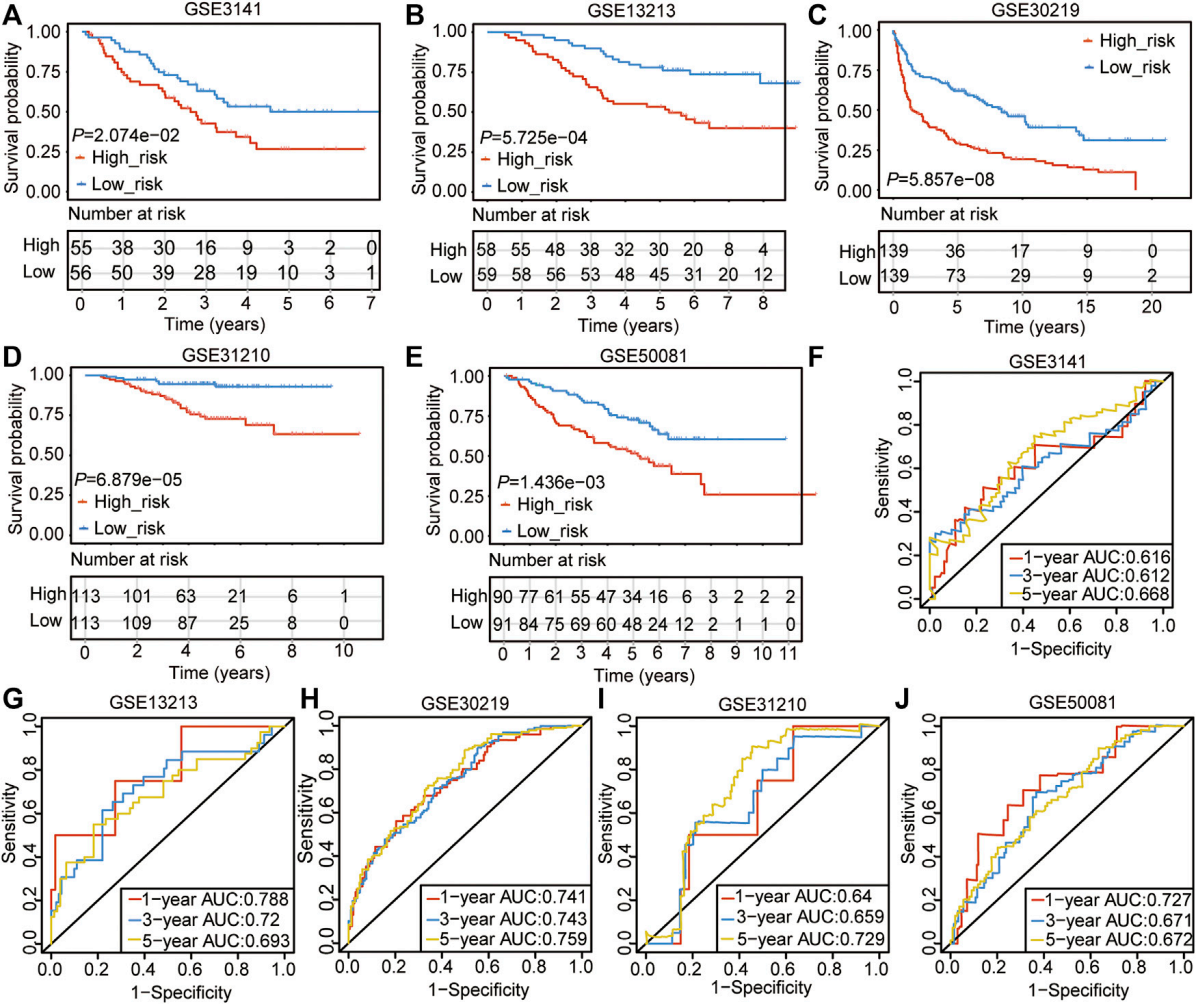
Validation of the CSRs-related prognostic signature with GEO datasets. **(A–E)** The overall survival of the high- and the low-risk group in the five validation sets. **(F–J)** ROC analyses of the cox model for 1-, 3-, and 5-years in the five validation sets.

We used the nine genes to conduct the Cox model in five GEO data sets (GSE3141, GSE13213, GSE30219, GSE31210, and GSE50081), which contains 913 LUAD samples (Table 3, Supplementary Table S12). The Kaplan-Meier curve showed the patients in the high-risk group had poor outcomes in the five validation sets (Figures 4A–E). The AUC value of 1-, 3-, and 5-years in the five validation sets were ranging from 0.612 to 0.788 (Figures 4F–J), which verified our previous results that the 9-gene prognostic signature was linked to predicting the OS of LUAD patients.

**TABLE 3:** Information of GEO data sets used in the validation of the Cox.

Gene Name				
	GPL6480	117 (49/68)	58 (33/25)	59 (16/43)
GSE31210	GPL570	226 (35/191)	113 (28/85)	113 (7/106)
GSE30219	GPL570	278 (188/90)	139 (115/24)	139 (72/66)
GSE3141	GPL570	111 (58/53)	55 (33/22)	56 (25/31)
GSE50081	GPL570	181 (75/106)	91 (48/42)	90 (27/64)

### Identification of Different Clusters Mediated by the 48 CSRs in LUAD.

**FIGURE 5:**
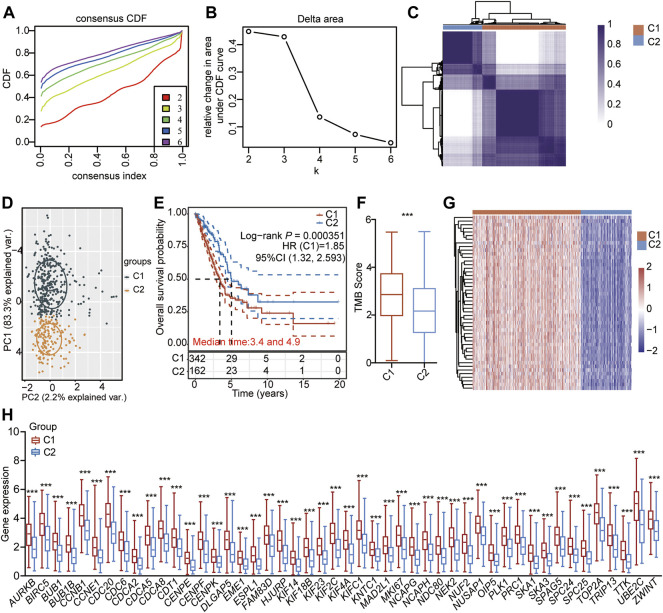
Unsupervised consensus clustering of LUAD-samples based on the 48 CSRs. **(A)** Consistent cumulative distribution shows the cumulative distribution function with different values of k, which is used to judge the optimal value of k. **(B)** Delta area map. **(C)** The consistency matrix of all data sets with k = 2. **(D)** PCA showing the LUAD-samples were divided into two distinct clusters. **(E)** The OS of LUAD-patients in the cluster 1 and 2. **(F)** The difference in TMB between the two clusters. The significance was calculated by Wilcoxon rank-sum test. ^***^ *p* < 0.001. **(G)** Heatmap showing the expression pattern of the 48 CSRs in the two clusters. **(H)** The 48 CSRs were all highly expressed in cluster 1. The significance was calculated by Wilcoxon rank-sum test. ^***^ *p* < 0.001.

To investigate the specific function of the CSRs involved in the development of LUAD, the unsupervised consensus clustering analysis was conducted for LUAD samples based on the expression of 48 CSRs. The result showed that the 497 LUAD samples were divided into two distinct subgroups of cluster 1 (C1, *n* = 338) and cluster 2 (C1, *n* = 159) (Figures 5A–D, Supplementary Table S13). The Kaplan-Meier curve showed that patients in C1 had a worse outcome than those in C2 (*p* = 0.000351, Figure 5E). The difference in TMB score between the two clusters was assessed, and the result showed that C1 had a higher TMB score (Figure 5F). Moreover, the 48 CSRs were consistently highly expressed in group C1 compared with those in cluster 2 (Figures 5G,H, Supplementary Table 14), validating the existence of distinguishing CSRs patterns in LUAD, and the cluster 1 may represent high-risk groups.

### DEGs and the Functional Analysis Between the Two CSRs-Related Clusters

**FIGURE 6:**
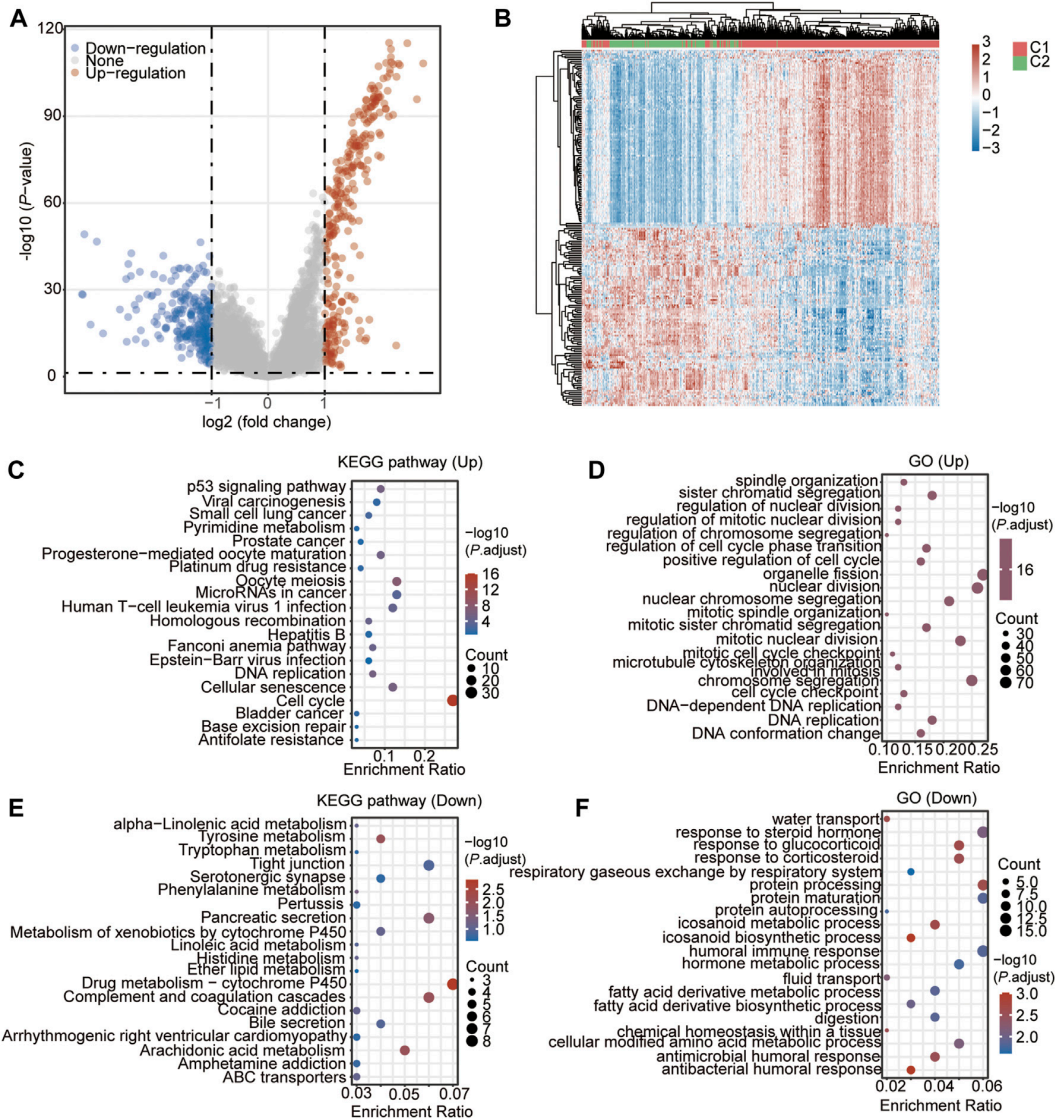
DEGs and the functional analysis between the two CSRs-related clusters. **(A)** Volcano Plot showing the DEGs in cluster 1 vs. cluster 2. **(B)** Heatmap showing the DEGs in cluster 1 vs. cluster 2. **(C)** KEGG enrichment of up-regulated genes in cluster 1. **D** GO enrichment of up-regulated genes in cluster 1. **(E)** KEGG enrichment of down-regulated genes in cluster 1. **(F)** GO enrichment of down-regulated genes in cluster 1.

Then, we identified the DEGs between the two clusters. As a result, a total of 536 genes (278 up-regulated genes and 258 down-regulated genes) were found to be significantly and differently expressed in group C1 compared to group C2 (Figures 6A,B, Supplementary Table S15). The subsequent KEGG and GO showed that the up-regulated genes were enriched in the pathways of cell cycle (Figure 6C, Supplementary Table S16), and the biological processes of chromosome segregation, organelle fission, and nuclear division, and mitotic nuclear division (Figure 6D, Supplementary Table S16). The down-regulated genes were enriched in the pathways of Complement and coagulation cascades, Arachidonic acid metabolism, and Drug metabolism–cytochrome P450 (Figure 6E, Supplementary Table S16), and biological processes of antibacterial humoral response, eicosanoid biosynthetic process, protein processing, respond to corticosteroid, respond to glucocorticoid, and eicosanoid metabolic process (Figure 6F, Supplementary Table S16).

### Identification of Hub-Genes Between the Distinct Clusters

**FIGURE 7:**
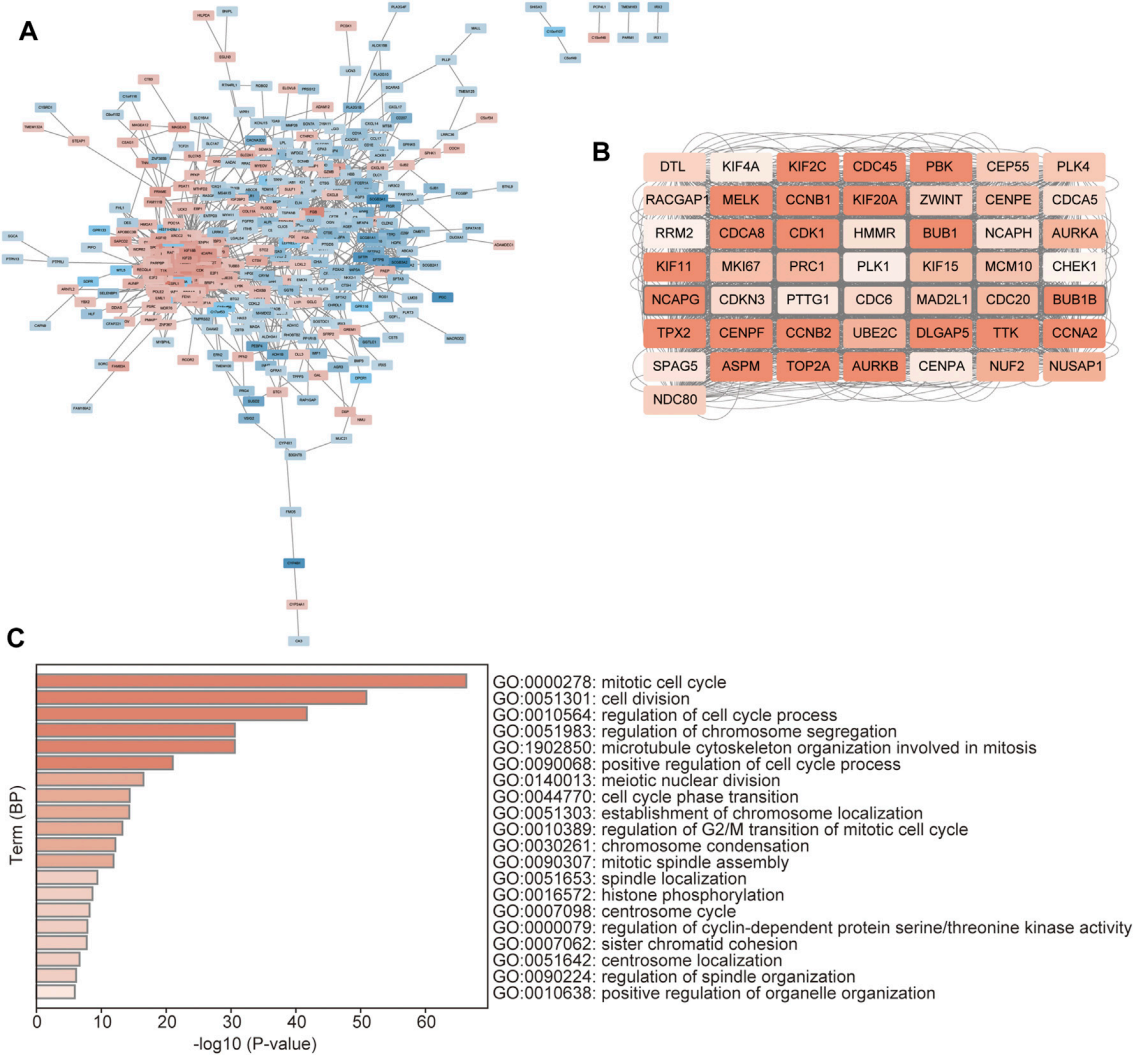
Identification of hub-genes between the distinct clusters. **(A)** PPI network of the DEGs between the two clusters. The red represents the up-regulated genes, and the blue represents the down-regulated genes. **(B)** The network of the top 50 hub genes. The color depth represents the rank of the genes. **(C)** GO enrichment of the hub genes, which was performed by the Metascape database (https://metascape.org/gp/index).

The 536 DEGs between the two CSRs-related clusters were used to perform the PPI network, which showed that there was an obvious group with a close correlation occurring in the up-regulated genes (Figure 7A). We calculated the rank by cytohubba in the PPI network, and the top 50 hub-genes were selected, which were all up-regulated genes in cluster 1 (Figure 7B, Supplementary Table S17). The subsequent GO showed these genes were enriched in the biological processes of the mitotic cell cycle, cell division, and regulation of chromosome segregation (Figure 7C).

### CSRs Are Associated With Immunization Checkpoint Block in LUAD

**FIGURE 8:**
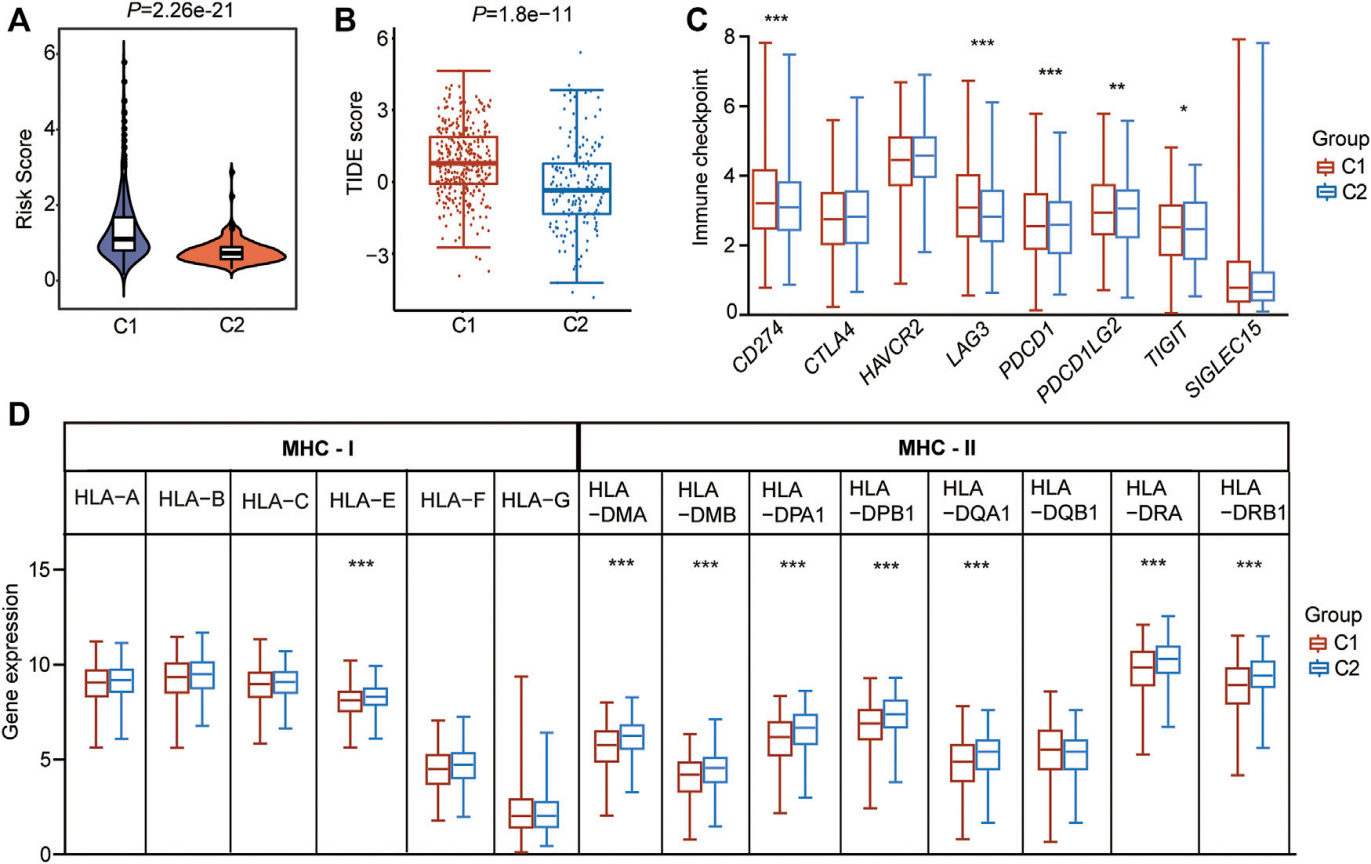
CSRs are associated with immunization checkpoint block in LUAD. **(A)** The risk score in the two clusters. **(B)** The TIDE score in the two clusters. **(C)** The immune checkpoint genes in the two clusters. **(D)** The HLA family genes in the two clusters. The statistical significance was calculated *via* Wilcoxon rank-sum test, ^***^ *p* < 0.001, ^**^ *p* < 0.01, ^*^ *p* < 0.05.

Cancer cells must have evaded the anti-tumor immune response to grow progressively, which relies in part on the expression on their surface of proteins with immunosuppressive functions, such as programmed cell death 1 (PD-L1) (Reisländer et al., 2020). Enhanced ability to escape immune detection always caused malignant development of cancer cells, and the following poor outcome for patients with LUAD. To find out the possible molecular mechanism that the CSRs impact on the prognosis, the difference in immunization checkpoint block (ICB) score between the two clusters, including the levels of immune checkpoint genes, tumor immune dysfunction and exclusion (TIDE) score, and HLA component expression, were evaluated. We found that the C1 had a higher level of risk score (*p* = 2.26e-21, Figure 8A), and TIDE score (*p* = 1.8e-11, Figure 8B). Additionally, the immune checkpoint genes, CD274 (PD-L1), lymphocyte activating 3 (LAG3), programmed cell death 1 (PDCD1 (PD-1)), and programmed cell death 1 ligand 2 (PDCD1LG2), were significantly expressed higher in C1 than in C2 (Figure 8C). Moreover, among the eight MHC-Ⅱ components, seven molecules were expressed lower in C1 than in C2 (Figure 8D). One MHC-Ⅰ component was also expressed lower in C1 (Figure 8D). These all indicated that the patients in cluster 1 may have a higher immunization checkpoint block (ICB).

### CSRs Are Associated With Immune Characteristics of LUAD

**FIGURE 9:**
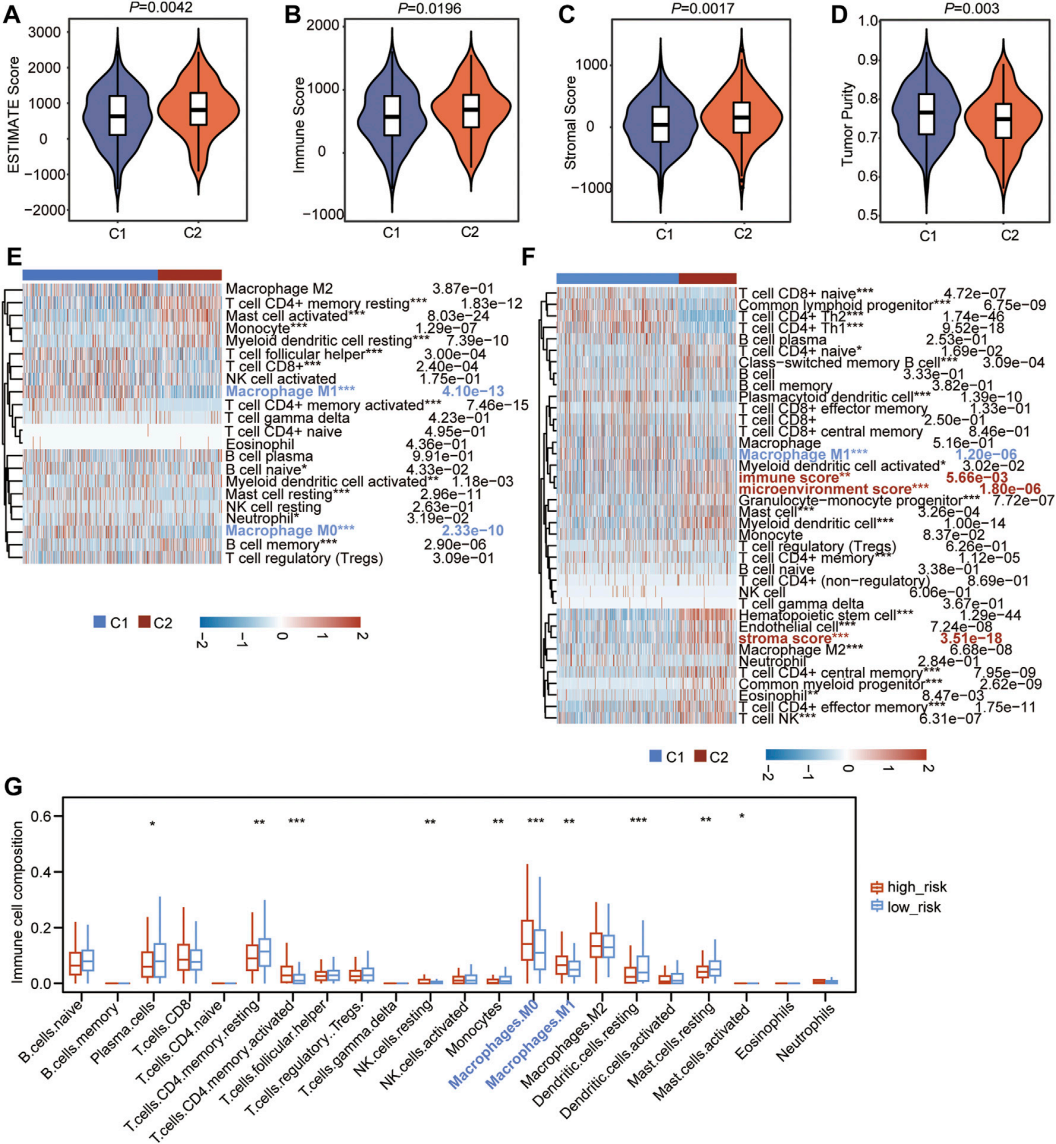
CSRs are associated with immune characteristics of LUAD. **(A–D)** Comparison of estimate score **(A)**, immune score **(B)**, stromal score **(C)**, and tumor purity **(D)** in the two clusters. **(E)** Heatmap showing the 22 types of immune cells infiltration in the two clusters by CIBERSORT algorithm. **(F**) Heatmap showing the 27 types of immune cells infiltration in the two clusters by xCell algorithm. The red label represents the cells that were highly infiltrated in cluster 2, the blue label represents the cells that were highly infiltrated in cluster 1. The statistical significance was calculated via Wilcoxon rank-sum test, ^***^ *p* < 0.001, ^**^ *p* < 0.01, ^*^ *p* < 0.05. **(G)** The difference in infiltrated abundance of immune cells between the two risk groups. The infiltrated abundance of immune cells was calculated by CIBERSORT algorithm. The statistical significance was calculated via Wilcoxon rank-sum test, ^***^ *p* < 0.001, ^**^ *p* < 0.01, ^*^ *p* < 0.05.

To further explain the possible impact of the CSRs on anti-tumor immune response, the TME characteristics between the two clusters were also assessed. The result of TME showed that the ESTIMATE score (*p* = 0.0042), immune score (*p* = 0.0196), and stromal score (*p* = 0.0017) were lower in cluster 1 than in cluster 2 (Figures 9A–C), while the tumor purity (*p* = 0.003) was higher in C1 than in C2 (Figure 9D). CIBERSORT algorithm was firstly used to calculate the 22 types of immune cells. The result showed that the filtrating proportion of resting memory CD4^+^ T cells, activated mast cells, resting myeloid dendritic cells, and memory B cells were higher in cluster 2 than in cluster 1 (Figure 9E), while the filtrating proportion of follicular helper T cells, CD8^+^ T cells, M0 macrophages, and M1 macrophages were higher in cluster 1 than in cluster 2 (Figure 9E). The xCELL algorithm was further used to assess another immune cell set, which contains 35 kinds of immune cells. The result showed that the proportion of T cell CD8^+^ naïve, Common lymphoid progenitor, CD4^+^ Th2, CD4^+^ Th1, plasmacytoid dendritic cell, and M1 macrophage were higher in cluster 1 (Figure 9F), while the content of activated myeloid dendritic cell, granulocyte-monocyte progenitor, mast cell, myeloid dendritic cell, M2 macrophage, memory CD4^+^ effector T cells, and NK T cells were higher in cluster 2 (Figure 9F). Besides this, immune score, microenvironment score, and stroma score were lower in cluster 1 than in cluster 2, which was consistent with our previous result (Figure 9F).

The difference in the abundance of 22 immune microenvironment infiltrating cells calculated by CIBERSORT algorithm between high- and low-risk groups in TCGA cohort and two GEO datasets was further revealed. The result in TCGA cohort showed that the high-risk group has a very similar pattern of infiltrated immune cells as the C1 population (Figure 9G). In addition, the infiltrating abundance of M0 and M1 macrophages were significantly higher in the high-risk group in all three datasets (Figure 9G, Supplementary Figure S2), and the filtrating proportion of resting memory CD4^+^ T cells was lower in the high-risk group compared to the low-risk group in all three datasets (Figure 9G, Supplementary Figure S2), indicating that the nine-gene independent prognostic signature was positively correlated with the infiltrating abundance of M0 and M1 macrophages and negatively associated with resting memory CD4^+^ T cells. These all demonstrated that the CSRs were associated with the immune characteristics in the TME of LUAD.

### The Small-Molecular Chemotherapeutics Forecast for High-Risk Patients Based on the Hub-Genes

**FIGURE 10:**
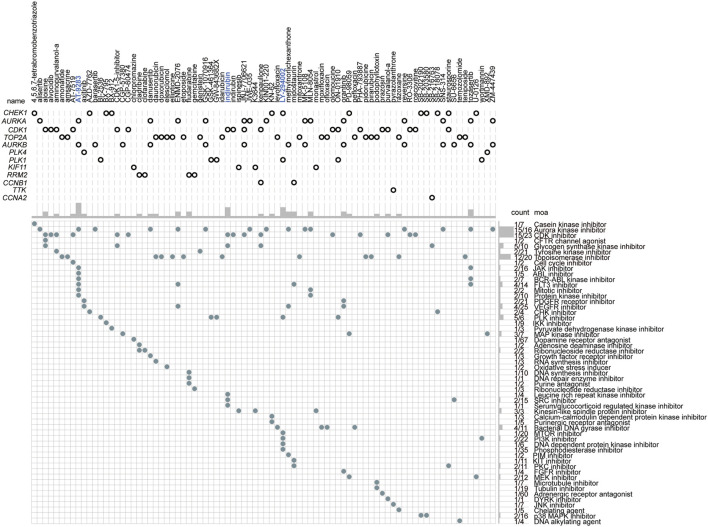
The small-molecular chemotherapeutics forecast for patients with high-risk based on the hub genes. The blue label represents the most outstanding small-molecular perturbagens.

Our previous results showed that patients in cluster 1 were the predicted high-risk population, and we then wanted to find the potential chemotherapeutics suited for these populations. According to the different biological characteristics between the two clusters, the hub genes were assumed to be the critical and potential targets for the therapy of the high-risk groups. Therefore, the adjuvant chemotherapeutics targeting the 50 hub genes were assessed through the mode of action (moa) module in the CMap database. The result showed that a total of 74 small-molecular perturbagens, targeting aurora kinase B (*AURKA*), cyclin-dependent kinase 1 (*CDK1*), *TOP2A*, *AURKB*, polo-like kinase 4 (*PLK4*), *PLK1*, ribonucleotide reductase regulatory subunit M2 (*RRM2*), *CCNB1*, *TTK*, and cyclin A2 (*CCNA2*), were predicted to be the potential chemotherapeutics for patients in cluster 1 (Figure 10). A total of 47 moas were predicted to be the possible pathways by which these chemotherapeutics function, such as Aurora kinase inhibitor, CDK inhibitor, and topoisomerase inhibitor (Figure 10). Among the 74 potential chemotherapeutics, AT-9283, indirubin, and LY-294002 were the most outstanding ones, which function as the most common pathways (marked in blue, Figure 10).

## Discussion

Lung cancer is the most common malignancy and remains the leading cause of cancer mortality worldwide (Bade and Cruz, 2020). Approximately 85% of patients have a histological subtype known as non-small cell lung cancer (NSCLC) (Herbst et al., 2018), with the main subtype of LUAD (Herbst et al., 2018; Sun and Zhao, 2019). Recently, LUAD has been the major cause of cancer-associated mortality with a poor prognosis (Song et al., 2021). Chromosomal abnormalities are reported to be a common characteristic of cancer, which is attributed to the ongoing chromosome segregation errors during mitosis (Bakhoum et al., 2018; Kou et al., 2020a). It is associated with poor outcomes and therapeutic resistance in various cancers (Potapova and Gorbsky, 2017; Bakhoum and Cantley, 2018; Kou et al., 2020a). Chromosomal segregation errors or the alteration of CSRs are usually the critical factors of genomic instability that drive tumor evolution (Hanahan and Weinberg, 2011; Bolhaqueiro et al., 2019; Tayoun et al., 2021). For example, aneuploidy is a direct consequence of chromosome segregation errors in mitosis (Janssen et al., 2011; Kawakami et al., 2019). This is always accompanied by chromosomal instability (CIN) and genomic instability and causes additional chromosome gains or losses in a significant proportion of cell divisions, which further manifests the complexity of cancer karyotypes (Dürrbaum and Storchová, 2016). Aneuploidy correlates with increased metastatic and drug resistance, indicating that Aneuploidy is more beneficial for cancerous cells than diploid cells (Dürrbaum and Storchová, 2016). The genomic diversification caused by chromosome segregation errors usually promotes tumor evolution and heterogeneity (Soto et al., 2019), which is an important reason for therapeutic resistance (Dagogo-Jack and Shaw, 2018; Lim and Ma, 2019). Although aneuploidy provides advantages for the proliferation and drug resistance of cancer cells, excessive aneuploidy beyond a critical level is lethal to cancer cells (Kawakami et al., 2019). Therefore, molecular and bioprocesses engaged in chromosome segregation should be utilized as potential therapeutic targets for cancers.

Previous studies have indicated that CIN and loss of heterozygosity (LOH) play significant roles in the development of LUAD (Ninomiya et al., 2006). In this study, chromosomal segregation during mitosis was found to be correlated with the poor prognosis of LUAD. Among the determined 2,416 prognosis-related genes, 128 genes were found to be enriched in the biological processes of chromosomal segregation, indicating that these chromosomal segregation-related genes may play an important role in the development of LUAD. 48 of 128 genes were found to be up-regulated in LUAD compared with the normal people and were associated with the high TMB in patients with LUAD. Besides, 47 genes were identified as the prognostic signatures after suffering from univariate cox regression. By LASSO and multivariate cox regression, nine genes were finally determined as the independent prognostic signature to construct the nomogram cox model. Patients in the high-risk subgroup had significantly poor OS. Notably, *PLK1* and *DLGAP5* were found to have valuable significance for the prediction of prognosis. PLK1 plays multiple roles in the initiation, maintenance, and completion of mitosis (Liu et al., 2017), maintaining genome stability (Zhang et al., 2016; Gheghiani et al., 2021), and DNA damage response (DDR) (Peng et al., 2021). It is found to be highly expressed in most of the human cancers (Gutteridge et al., 2016), and enhanced gene expression is associated with a poor prognosis (Ramani et al., 2015; Tut et al., 2015; Zhang et al., 2015; Chen Z. et al., 2019; Montaudon et al., 2020). Besides, PLK1 has been reported to be associated with chemotherapeutic drugs resistance, including doxorubicin (Wang P. et al., 2018), paclitaxel (Gasca et al., 2016; Shin et al., 2019; Shin et al., 2020), metformin (Shao et al., 2015; Zhu et al., 2022), and gemcitabine (Song et al., 2013; Li et al., 2016; Mao et al., 2018). DLGAP5 is a microtubule-associated protein and is identified to be a prognosis biomarker in various cancers (Schneider et al., 2017; Branchi et al., 2019; Xu et al., 2020; Zheng et al., 2020; Feng Y. et al., 2021; Zhou D. et al., 2021). Inhibition of this gene suppresses cell proliferation and invasion, induces G_2_/M phase arrest, and promotes apoptosis in human cancers (Liao et al., 2013; Zhang et al., 2021a). Not only these findings were confirmed, but also the predictive value of the two genes was proposed in this study. Here, *PLK1* and *DLGAP5* were found as the independent prognostic signatures combined with *HJURP*, *KIF14*, *OIP5*, *TTK*, *ESPL1*, *KIF18B*, and *NUF2* to predict the prognosis of LUAD-patients. *HJURP* is reported to be an oncogene that promotes cancer cell proliferation, migration, and invasion (Chen et al., 2018; Wei et al., 2019; Serafim et al., 2020; Li Y. et al., 2021; Lai et al., 2021). *KIF14* is confirmed to promote cancer cell proliferation and contribute to chemoresistance (Singel et al., 2014; Wang Z. Z. et al., 2018; Xiao et al., 2021). Silencing *TTK* is also found to inhibit the proliferation and invasion and increase radiosensitivity and chemosensitivity of cancer cells (Chen S. et al., 2019; Huang et al., 2020; Liu Y. et al., 2021; Zhang et al., 2021b; Qi et al., 2021). *ESPL1* is determined to be a novel prognostic biomarker and is associated with the malignant features in several cancers (Finetti et al., 2014; Wang R. et al., 2020; Liu Z. et al., 2021). *KIF18B* is also illustrated to be the oncogenesis to promote tumor progression and enhance therapeutic resistance (Li B. et al., 2020; Liu et al., 2020; Jiang J. et al., 2021). *OIP5* upregulation is observed in human cancers (Chow et al., 2021), and seems to be linked to drug resistance (Rodrigues-Junior et al., 2018). *NUF2* is found to be a prognostic biomarker and therapeutic target which is correlated with the immune infiltration in patients with cancer (Jiang X. et al., 2021; Shan et al., 2021; Xie et al., 2021). Combining the expression of the nine genes, we conducted the cox risk model. The subsequent DCA and calibration verified the accuracy of the model to predict the prognosis of LUAD. However, we found the nine-gene prognostic signature had no impact on the clinical pNTM stage of patients. This may be attributed to the small sample size, thus, larger samples should be analyzed further.

According to the unsupervised consensus clustering by the expression of the 48 CSRs, the 497 TCGA-LUAD samples were divided into clusters 1 and 2. The 48 CSRs were all highly expressed in cluster 1, and the risk score was significantly higher in cluster 1. The Kaplan-Meier showed that patients in cluster 1 had a poor OS. These all suggest that the patients in cluster 1 have a higher risk than those in cluster 2, and the subgroup cluster 1 is deemed to be the high-risk group. A total of 536 genes (278 up-regulated genes and 258 down-regulated genes) were determined to be the DEGs in cluster 1 compared to cluster 2. The up-regulated genes were enriched in the pathways of cell cycle and the biological processes of chromosome segregation, organelle fission, and nuclear division; the down-regulated genes were enriched in the pathways of drug metabolism-cytochrome P450 and the biological process of protein processing. The main function of cytochrome P450 (CYP450) is oxidative catalysis of endogenous and exogenous substances (Mittal et al., 2015). 80% of drugs currently in use, including anti-cancer drugs, are involved in phase I metabolism of CYP450 (Mittal et al., 2015). Interestingly, the hub genes with top 50 ranks were all highly expressed in cluster 1, which were enriched in the biological processes of mitotic cell cycle, regulation of chromosome segregation, and microtubule cytoskeleton organization involved in mitosis. As we discussed previously, the dysregulation of mitotic chromosome segregation is associated with poor prognosis and therapeutic resistance in human cancers. Among the 50 hub genes, *AURKB*, *BUB1*, *BUB1B*, *CCNB1*, *CDCA8*, *CENPF*, *DLGAP5*, *KIF2C*, *NCAPG*, *TOP2A*, *TTK*, assembly factor for spindle microtubules (*ASPM*), cyclin A2 (*CCNA2*), cyclin B2 (*CCNB2*), cell division cycle 45 (*CDC45*), cyclin-dependent kinase 1 (*CDK1*), kinesin family member 11 (*KIF11*), kinesin family member 20A (*KIF20A*), maternal embryonic leucine zipper kinas (*MELK*), PDZ binding kinase (*PBK*), TPX2 microtubule nucleation factor (*TPX2*) were found to be the hub genes with the rank 1, that were all reported to have pro-metastatic effects in human cancers (Chen et al., 2015; Bertran-Alamillo et al., 2019; Gan et al., 2019; Huang et al., 2019; Kou et al., 2020b; Wang L. et al., 2020; Wang X. et al., 2020; Hu et al., 2021; Wang S. et al., 2021; Li M.-X. et al., 2021; Wu et al., 2021; Feng Z. et al., 2021; Huang et al., 2021).

The difference in ICB between the two clusters was further assessed, including TIDE score, immune checkpoint genes, HLA family components to evaluate the possible anti-tumor immune response associated with the CSRs. The cluster 1 subtype featured lower levels of HLA family genes, higher TIDE scores, and higher levels of immune checkpoint genes, indicating that patients in cluster 1 have the higher ICB and, in turn, a possible poor anti-tumor immune response. The highly expressed immune checkpoint genes, *CD274*, *LAG3*, *PDCD1*, *PDCD1LG2*, in this subgroup further confirmed the result. PDCD1 (PD-1) is a key coinhibitory receptor expressed on activated T cells (Ai et al., 2020). The engagement with its ligands, mainly PD-L1, leads to the events of inhibition of T cell proliferation, activation, cytokine production, alters metabolism and cytotoxic T lymphocytes (CTLs) killer functions, and eventual death of activated T cells (Ai et al., 2020). The overexpression of PD-L1 has been verified to contribute to the immune surveillance evasion of cancer cells and caused the invasion and migration (Iwai et al., 2002). PDCD1LG2 (PD-L2) is a second ligand for PD-1 and inhibits T cell activation (Latchman et al., 2001). LAG3 (CD223), an emerging targetable inhibitory immune checkpoint molecule, is the third inhibitory receptor pathway to be targeted in the clinic (Solinas et al., 2019). It is mainly found on activated immune cells and is involved in the exhaustion of T cells in malignant diseases (Puhr and Ilhan-Mutlu, 2019). LAG3 has been reported to play a negative regulatory role in cancer immunology by interacting with its ligands (Wang M. et al., 2021). What’s more, seven MHC-Ⅱ and one MHC-Ⅰ molecules were found to be under-expressed in cluster 1, further supporting the result that patients in cluster 1 have a poor antitumor immune response. MHC-Ⅰ molecules function to bind the encoded peptides, transport and display the antigenic information on the cell surface, and allow CD8^+^ T cells to identify pathological cells, such as cancers that are expressing mutated proteins (Dhatchinamoorthy et al., 2021). Loss of MHC-Ⅰ antigen presentation always leads to cancer immune evasion (Dhatchinamoorthy et al., 2021). MHC-Ⅱ is an antigen-presenting complex, that is important for antigen presentation to CD4^+^ T cells. Tumor-specific MHC-Ⅱ is associated with favorable outcomes in patients with cancer, including those with immunotherapies (Axelrod et al., 2019). The lower level of MHC-Ⅱ and one MHC-Ⅰ molecule in patients of cluster 1 may help the tumor evasion of immune checkpoints and poor immunotherapies. What’s more, we found a higher TMB score in cluster 1, which was positively associated with the 48 CSRs. A recent study has shown that the average copy number variation (CNVA) of chromosome fragments is a potential surrogate for tumor mutational burden in predicting responses to immunotherapy in NSCLC (Lei et al., 2021). Therefore, the increased genomic instability in tumors with dysregulated chromosome segregation alters mutational load and in turn impacts antitumoral immune responses, which further impacts the prognosis in LUAD. However, we cannot define either CIN was a cause or result of somatic mutation, and this needs more experiment assays to be determined.

Meanwhile, the infiltrating proportions of immune cells were further evaluated to analyze the difference in immune characteristics between the two clusters. We found that cluster 1 had a lower immune, stromal, and ESTIMATE score compared with cluster 2. According to the CIBERSORT and xCell algorithms, cluster 1 was shown to have a higher infiltrating proportion of M0 and M1 macrophages, while cluster 2 had a higher infiltrating proportion of M2 macrophages and mast cells. Macrophages play a critical role in cancer development and metastasis, which could be identified as 2 major subpopulations of M1 macrophages (Proinflammatory) and M2 macrophages (anti-inflammatory) (Xia et al., 2020). M0 macrophages are naïve macrophages without polarization. M1 macrophages have antimicrobial and antitumoral activity, while M2 macrophages participate in angiogenesis, immunoregulation, tumor formation, and progression (Shapouri-Moghaddam et al., 2018). Increased mast cell density is associated with the prognosis and plays a multifaced role in TME by regulating tumor biology, including cell proliferation, angiogenesis, invasiveness, and metastasis (Aponte-López and Muñoz-Cruz, 2020). These results seem contradictory that M1 macrophages have antitumoral activity but were higher infiltrated in cluster 1, whereas M2 macrophages have protumoral activity but were lower infiltrated in cluster 1, as were the follicular helper T cells and CD8^+^ T cells. We think this elevation may be a compensatory effect, that these antitumoral immune cells are regulated to increase against the tumor cells. However, the effective activity of these anti-tumor immune cells may be inhibited because of the high levels of the immunosuppressive proteins on their surface. Most tumor-infiltrating immune cells were functionally inactive.

Based on the 50 hub genes that were upregulated in cluster 1, a total of 74 small-molecule perturbagens were predicted to be the potential chemotherapeutics that were possibly suited for these patients at high risk. Among these, AT-9283, indirubin, and LY-294002 were the most outstanding ones, which function as the most common pathways. A total of 47 moas, including Aurora kinase inhibitor, CDK inhibitor, and topoisomerase inhibitor, were forecasted to be the possible molecular mechanism by these chemotherapeutics functions. The inhibitory role of AT-9283 in cancer cell growth and survival has been demonstrated in cell-based systems (Kimura, 2010). Although phase Ⅱ clinical trials have not been completed, it showed good safety and efficacy in phase Ⅰ clinical trials conducted in patients with hematological malignancies and solid tumors (Kimura, 2010). Indirubin has also been reported to exert anticancer effects in human cancers (Li Z. et al., 2020). LY-294002 enhances the chemosensitivity of liver cancer to oxaliplatin (Xu et al., 2021). We found here these small-molecule perturbagens might have favorable therapeutic effects for patients in cluster 1, who had the high expression of their target genes. This will provide an effective therapeutic regimen for the individual treatment in LUAD.

Our study is the first one to systematically analyze the relationship between chromosome segregation regulation and the immune microenvironment. This can provide a new direction for immune-related LUAD pathogenesis and therapeutic research. However, there are certain limitations in this current study. First, although the exploration of a decent number of samples and summarizing data can be helpful in the research community, this analysis may have some bias due to the small size of TCGA-LUAD cohort. The relationship between the prognostic signatures and clinical indices, such as pNTM staging, has not been well explained. Therefore, further analysis with larger samples is still needed. Second, further experimental verifications are necessary to elucidate the potential impact of these predicted genes in the immune microenvironment. Moreover, the protein expression levels of the hub CSRs in pathogenesis and progression of LUAD depend on further experimental studies to elucidate. Additionally, some of the genes we focused on in this study have been reported to be activated by post-translational modification and function as kinase. Therefore, database analyses depending on the gene expression profile at the mRNA levels have a limit. Further analysis should be focused on the protein or post-translational modification levels.

In conclusion, this study demonstrated that the CSRs were important factors to influence the development and progression of LUAD. The high expression of these regulators was correlated with the poor prognosis and the possible immunotherapeutic resistance in LUAD, which could be the potential therapeutic target for LUAD.

## Data Availability

The datasets presented in this study can be found in online repositories. The names of the repository/repositories and accession number(s) can be found in the article/Supplementary Material.
