# Supplementary material for: Chromatin Separation Regulators Predict the Prognosis and Immune Microenvironment Estimation in Lung Adenocarcinoma
Source: Front Genet. 2022 Jul 8;13:917150. doi: 10.3389/fgene.2022.917150 (PMC9305311; doi:10.3389/fgene.2022.917150)
Supplement: Supplementary file 1 [file DataSheet-1.zip › Supplementary materials/Supplementary Figure.docx]

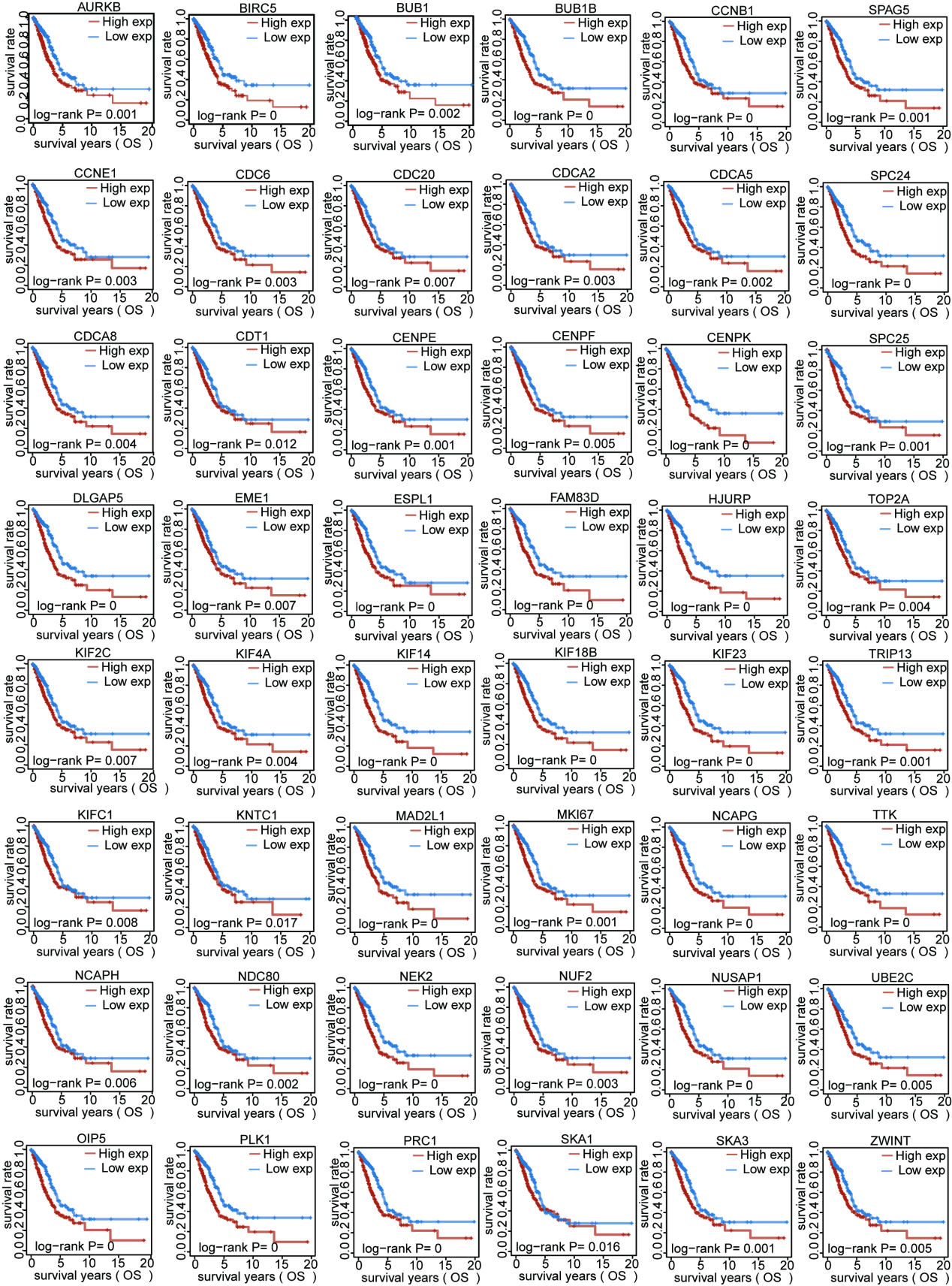


**Supplementary Figure 1 The OS of the 48 CSRs in LUAD.**

**
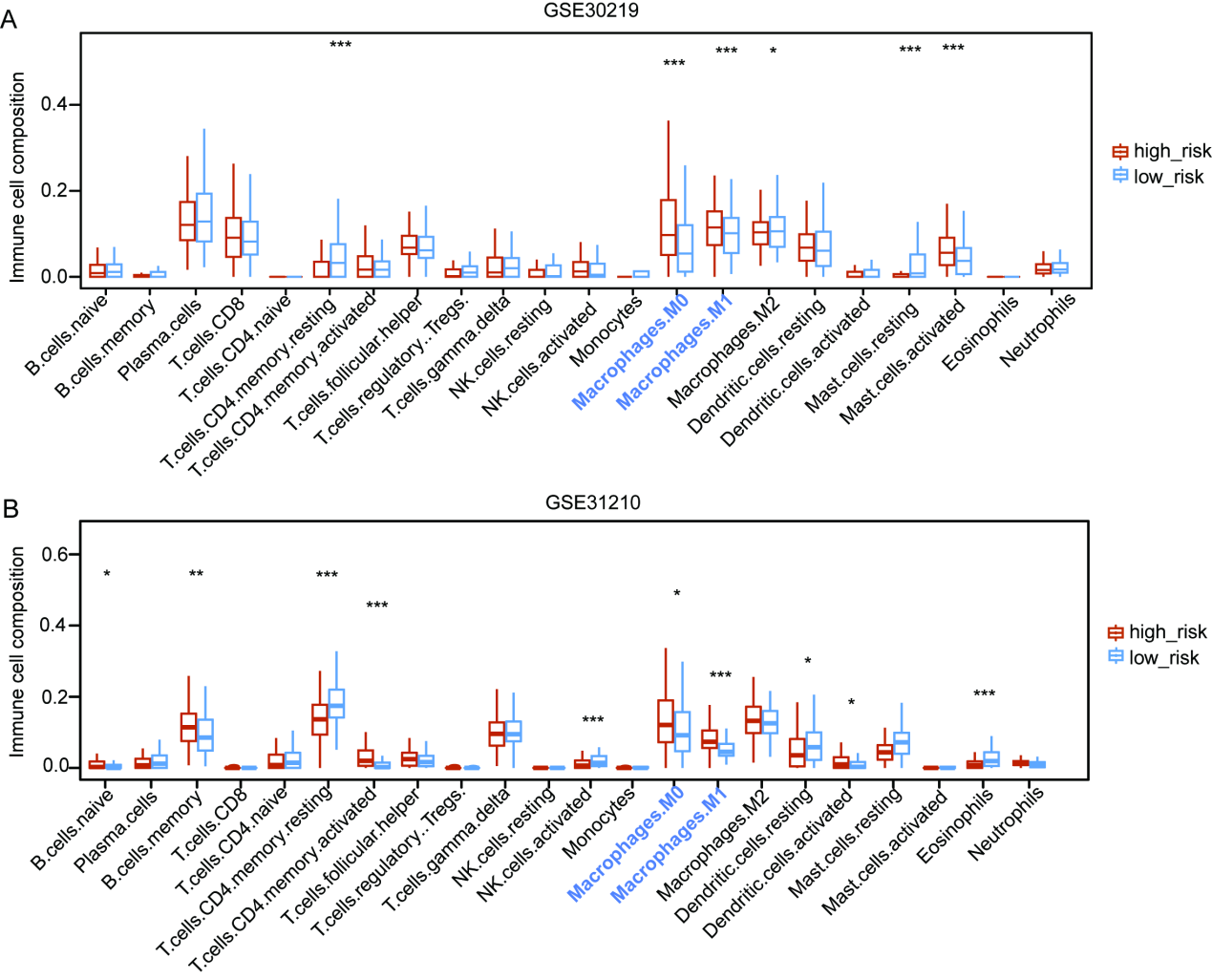
**

**Supplementary Figure 2** **The immune cell infiltration proportion in the two risk groups. A-C** The box plots showing the difference in the infiltrating proportion of immune cells between the two risk groups respectively in GSE30219 (A) and GSE31210 (B) datasets. The infiltrating proportion of immune cells in TME was calculated by CIBERSORT algorithm. The significance was calculated by unpaired t-test, ****P*<0.001, ***P*<0.01, **P*<0.05.
